# Implication of MAPK, Lipocalin-2, and Fas in the protective action of liposomal resveratrol against isoproterenol-induced kidney injury

**DOI:** 10.1016/j.jsps.2023.101907

**Published:** 2023-12-10

**Authors:** Ahlam M. Alhusaini, Samiyah M. Alshehri, Wedad S. Sarawi, Hanan K. Alghibiwi, Sumayya A. Alturaif, Reema A. Al khbiah, Shog M. Alali, Shaikha M. Alsaif, Ebtesam N. Alsultan, Iman H. Hasan

**Affiliations:** a Department of Pharmacology and Toxicology, College of Pharmacy, King Saud University, P.O Box 22452, Riyadh 11495, Saudi Arabia; b Pharm D Program, College of Pharmacy, King Saud University, P.O Box 22452, Riyadh 11495, Saudi Arabia; c Department of Pharmaceutics, College of Pharmacy, King Saud University, P.O Box 22452, Riyadh 11495, Saudi Arabia; d Department of Pharmacognosy, College of Pharmacy, King Saud University, P.O Box 22452, Riyadh 11495, Saudi Arabia

**Keywords:** L-resveratrol, Isoproterenol, Cystatin c, MAPK, Fas, Lipocalin-2

## Abstract

**Background and Objective:**

Isoproterenol (ISO) is a non-selective β-adrenergic receptor agonist. It can be used to treat bradycardia and cardiogenic shock. Despite its usefulness, the overstimulation of β-receptors by ISO can cause “cardiorenal syndrome,” a term used to describe heart and kidney damage. Resveratrol (RES), a natural polyphenol, has marked anti-inflammatory and antioxidant activities. The present work was designed to study the protective efficacy of liposomal resveratrol (L-RES) against ISO-induced kidney injury.

**Materials and Methods:**

The kidney injury was induced in rats by administering ISO (50 mg/kg, s.c.) twice a week for 2 weeks. RES and L-RES were administered at a dose (20 mg/kg/ day, p.o.) along with ISO for 2 weeks. Inflammatory and apoptotic biomarkers were analyzed, which were validated using histochemical analysis.

**Results:**

ISO caused renal dysfunction, which manifested as elevated urea, creatinine and uric acid, besides cystatin c and MAPK protein overexpression. In addition, ISO induced gene expression of Fas and lipocalin-2 and provoked genomic DNA fragmentation in renal tissues as compared with the control group. Histological examination confirmed morphological alterations of the kidney tissues obtained from the ISO group. Concurrent treatment of either RES or L-RES with ISO significantly ameliorated kidney damage as demonstrated by the improvement of all measured parameters with the best results for L-RES. The histopathological findings were correlated with the above biochemical parameters.

**Conclusion:**

L-RES could be a promising approach for the prevention of kidney injury induced by ISO, most likely via the downregulation of MAPK, cystatin c, Fas, and lipocalin-2.

## 1 Introduction

Isoproterenol (ISO), a synthetic catecholamine, is a β-1and β-2 adrenergic receptor agonist structurally similar to adrenaline. Through this mechanism, it causes an increase in heart rate and contractility, while in the kidney, it activates renin-angiotensin-aldosterone system (Desimine et al. 2018). American Heart Association (AHA) has only approved the use of ISO in heart block and cardiac arrest when pacemaker therapy is unavailable (Van Diepen et al. 2017). On the other hand, there are many off-labeled uses of ISO, such as bradycardia, cardiogenic shock, torsade de pointes, and bradycardia in cardiac transplant patients (Neumar et al. 2010). Despite its effectiveness, overstimulation of β-receptors, especially β-1 by ISO, can cause cardiorenal syndrome, a term used to describe the damage to both heart and kidneys. Acute or chronic heart dysfunction causes kidney dysfunction and vice-versa (Ghartavol et al. 2019). There is a link between renal dysfunction and myocardial infarction (MI) (Anamalley et al. 2022). Administration of an overdose of ISO was reported to cause MI in rats, which was linked to myocardial hyperfunction due to the imbalance between the oxygen supply and demand (Emran et al. 2021) which can eventually lead to cardiac and renal failure (Anamalley et al. 2022).

In animal model, ISO has been used as a remarkable initiator of kidney damage, which is correlated with the generation of free radical and inflammation and lead to cell death/apoptosis (Selim et al. 2021). The endogenous antioxidant components play a critical role in counteracting the oxidative stress progression; however, the excessive production of the reactive oxygen species (ROS) reduces the internal antioxidant capacity, causing multiple organ failure. Hence, there is a necessity to establish an antioxidant agent to counteract ROS overproduction and prevent disease development.

Resveratrol (RES) is a natural polyphenolic compound synthesized by plants mainly found in soy, vegetables, and fruits. RES has many benefits to human health. It possesses antioxidant, anti-inflammatory and other biological properties (Salehi et al. 2018). RES reduced renal injury by suppressing oxidative stress, lipid peroxidation, and inflammation (Chowdhury et al. 2022). Since RES has a short half-life and metabolizes rapidly, the liposomal delivery system has been introduced to reduce these limitations (Vanaja et al. 2013). Liposomal form of RES (L-RES) is a promising protective strategy as it enhances the RES bioavailability and solubility (Alhusaini et al. 2021). Therefore, in the present study, we investigated the protective efficacy of L-RES on kidney injury triggered by ISO, focusing on the modulation of The mitogen-activated protein kinases (MAPK), cystatin c, Fas, and lipocalin-2.

## 2 Materials and methods

### 2.1 Drugs and chemicals

RES raw powders were purchased from Sigma Chemical Co. (St. Louis, MO, USA), while ISO injection was obtained from a local pharmacy. L-RES was purchased from Lipolife, UK. L-RES has a particle size less than 200 nm with a neutral zeta potential as stated by the manufacturer. Urea, creatinine and uric acid biochemical assays were supplied from Randox, Crumlin, UK. IL-6 ELISA kit was obtained from R&D Systems (Minneapolis, MN, USA). MAPK, cystatin c and β-actin antibodies were purchased from Novus Biologicals (Centennial, CO, USA). The PCR primers were obtained from Sigma (St. Louis, MO, USA).

### 2.2 Experimental animals and treatments

Thirty-two adult male Wistar rats weighing 150–180 g were obtained from the Animals Centre at the College of Pharmacy, King Saud University, Riyadh, Saudi Arabia. They were housed under standard laboratory conditions with free access to food and water. The experimental protocol was approved by the Scientific Ethics Committee at King Saud University (KSU-SE-22-111).

After one week of acclimation to laboratory conditions, rats were randomly assigned into four groups of 8 animals each. Normal control group: rats were given 1 % carboxymethylcellulose (CMC). ISO Group: rats received ISO at a dose of 50 mg/kg (s.c.) twice weekly for a duration of two weeks (Alam et al. 2018). RES and L-RES groups: rats were treated with either RES or L-RES (20 mg/kg/day, p.o.) dissolved in 1% CMC (Alhusaini et al. 2022). for 14 days along with ISO.

Later, rats were anesthesized using carbon dioxide and sacrificed by decapitation. Blood was collected for sera separation, and the kidneys were excised immediately. Sagittal cuts of the kidney tissues were fixed in 10% formalin for histopathological study. Some kidneys were immediately frozen in liquid nitrogen and stored at −80 °C for molecular analysis. The rest was homogenized (20% w/v) in phosphate buffer and the supernatant was collected and stored at −80 °C for biochemical analysis.

### 2.3 Renal function assays

The measurement of serum creatinine, urea and uric acid levels was conducted according to manufacturer instruction.

### 2.4 Determination of IL-6 level

Interleukin-6 (IL-6) was analyzed using a specific ELISA kit and the procedure was performed according to the manufacure’s instructions. The optical density of each well was determined by Biotek microplate reader at 450 nm.

### 2.5 Immunoblotting

Kidney tissue lysates were prepared to investigate the expression levels of cystatin c and MAPK. by homogenizing the tissue in 1x RIPA buffer supplemented with a phosphatase inhibitor in Qiagen TissueLyser. The lysates were spun for 5 min, the supernatants were combined with gel loading buffer and 2-mercaptoethanol, and then heated at 95 °C for 5 min. Equal protein concentration mixtures were then loaded into sodium dodecyl sulfate–polyacrylamide gel and allowed to separate the proteins based on their molecular weights. The gels were layered above the PVDF membrane in a Trans-Blot Turbo chamber. Following the blocking step, the membranes were incubated overnight with antibodies targeting cystatin c, MAPK and β-actin at 4 °C. Then, the membranes were subjected to three washes in TBST and probed with antirabbit HRP secondary antibodies for a duration of 1 h at room temperature. The signals were developed by Clarity™Western ECL Substrate and visualized ImageQuant LAS 4000 system. The quantification of band intensity was measured by ImageJ software and standardized to the loading control β-actin.

### 2.6 Gene expression assay

Changes in the gene expression of Fas and Lipocalin-2 were evaluated using the RT-PCR method. The total RNA was isolated by TRIzol reagent (ThermoFisher Scientific, Waltham, MA, USA), and RNA was quantified by nanodrop. The reverse transcription was performed using High Capacity cDNA Reverse Transcription kit (Applied Biosystems, Bedford, MA, USA). Then the amplification was performed by mixing cDNA, primers, nuclease-free water and SYBER™ Green PCR Master Mix (Applied Biosystems, Bedford, MA, USA). The cycling conditions were 95 °C for 10 min, 40 cycles at 95 °C for 15 s and 60 °C for 1 min. The obtained data were analyzed using the 2–ΔΔCt method and normalized to β-actin. The primer sequences are illustrated in Table 1.

**Table 1:** The primers used RT-PCR.

Gene and Accession No.	Primers (5′→ 3′)	PCR product size (bp)
Fas (NM_139194.2)	F: TGGAGTTGAAGAGGAGCGTTCGT R: TTCACCAGGCTGACACGGTTGA	98
Lipocalin-2 (NM_130741.1)	F: GGAATATTCACAGCTACCCTC R: TTGTTATCCTTGAGGCCCAG	211
β-actin (NM_031144.3)	F: AGGAGTACGATGAGTCCGGC R: CGCAGCTCAGTAACAGTCCG	71

### 2.7 DNA fragmentation

Agarose gel electrophoresis was used to assess the integrity of genomic DNA from kidney tissues. The results were presented as a percentage change of the control group.

### 2.8 Histopathological analysis

The fixed kidney sections (4-μm thicknesses) were dewaxed by xylene, followed by rehydration in a declining series of ethanol concentrations: 100%, 95%, and 70%, and finally placed in dH_2_O. The sections were then stained with hematoxylin and eosin (H&E). Subsequently, the slides were washed, dehydrated, mounted, and visualized by a light microscope.

### 2.9 Statistical analysis

GraphPad Prism 8 software was used to perform the statistical analysis. The differences across the research groups were evaluated by one-way analysis of variance (ANOVA) followed by Tukey’s post hoc analysis. A P-value ≤ 0.05 was considered statistically significant.

## 3 Results

### 3.1 RES or L-RES restored serum kidney function and attenuated inflammation in ISO-induced kidney injury

**Fig. 1:**
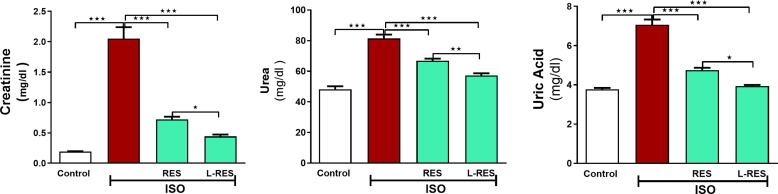
RES or L-RES prevented ISO-induced renal injury in rats. Treatments with RES or L-RES improved serum urea, creatinine, and uric acid levels, (n = 8). Data were expressed as mean ± SEM. *P < 0.05, **P < 0.01, ***P < 0.001.

Fig. 1 revealed that the serum kidney function tests (creatinine, urea, and uric acid) were elevated upon ISO administration as compared to the control group (p ≤ 0.001). Whereas treatment with the RES or L-RES declined almost of the previous distorted parameters compared to ISO- administration group (p ≤ 0.001). L-RES significantly reduced kidney function tests compared with its native compound.

**Fig. 2:**
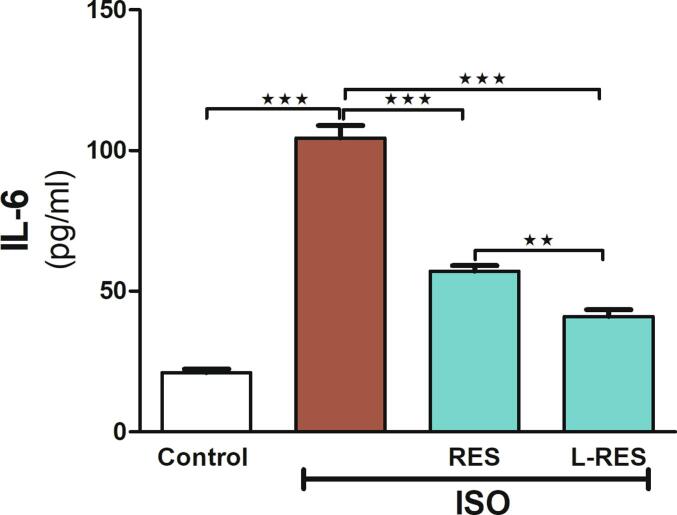
RES or L-RES attenuated IL-6 in ISO-induced renal injury in rats. Treatments with RES or L-RES attenuated serum IL-6 level, (n = 8). Data were expressed as mean ± SEM. *P < 0.05, **P < 0.01, ***P < 0.001.

Fig. 2 showed marked changes in serum levels of the inflammatory cytokines (IL-6) during ISO administration and after treatments. The use of ISO significantly increased IL-6 in comparison to the control group (p ≤ 0.001). On the contrary, the treatment of such antioxidants markedly declined the level of IL-6 compared to the ISO-administered group (p ≤ 0.001).

### 3.2 RES or L-RES diminished the ISO_-_induced renal toxicity via modulating protein expression of cystatin c and MAPK

**Fig. 3:**
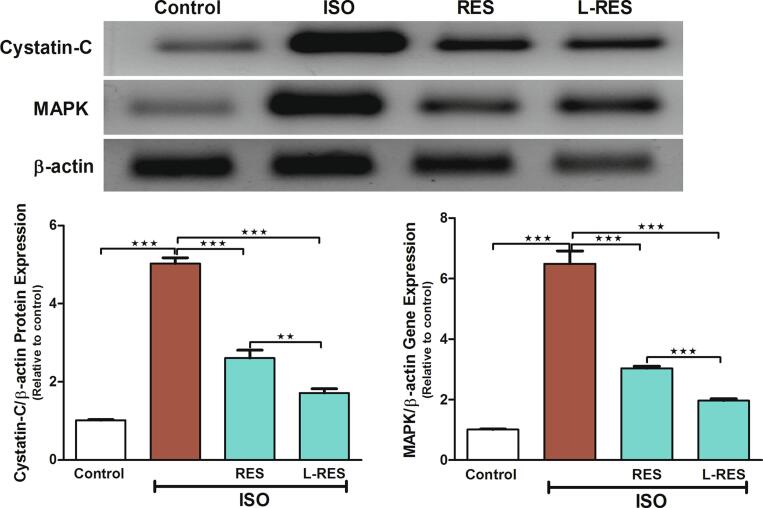
RES or L-RES downregulated the expression of renal MAPK and cystatin c. Representative blots showed changes in the expression of MAPK and cystatin c in all treated groups, (n = 8). Data were expressed as mean ± SEM. ***P < 0.001.

The effects of RES or L-RES on the protein expression of cystatin c and MAPK in the kidney tissues were determined by performing Western blotting (Fig. 3). The exposure to ISO caused a significant upregulation in cystatin c and MAPK (p ≤ 0.001) protein expression relative to the control group. Concurrent use of RES or L-RES significantly ameliorated ISO effects on these proteins (p ≤ 0.001) by restoring their regular expression levels. L-RES showed a further decrease in the expression of these proteins compared to RES treatment (p ≤ 0.001).

### 3.3 RES or L-RES regulated lipocalin-2 and Fas gene expression in ISO_-_induced renal toxicity

**Fig. 4:**
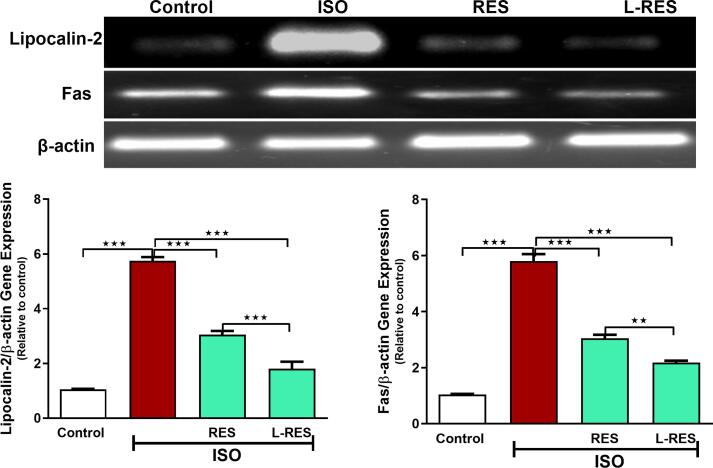
RES or L-RES downregulated the expression level of renal lipocalin-2 and Fas mRNA. A representative agarose image showed changes in the expression of lipocalin-2 and Fas genes in all treated groups, (n = 8). Data were expressed as mean ± SEM. **P < 0.01 and ***P < 0.001.

The effects of RES or L-RES on the gene expression of lipocalin-2 and Fas in the kidney of normal and toxic model rats were determined by RT-PCR (Fig. 4). ISO administration caused a significant upregulation in the expression levels lipocalin-2 and Fas (p ≤ 0.001) mRNA relative to the control group. RES or L-RES markedly ameliorated ISO effects on those genes (p ≤ 0.001) by restoring their levels. Using of L-RES showed a pronounced protective effect on the levels of those genes (p ≤ 0.01, p ≤ 0.001), respectively.

### 3.4 RES or L-RES prvented renal DNA damage induced by ISO

**Fig. 5:**
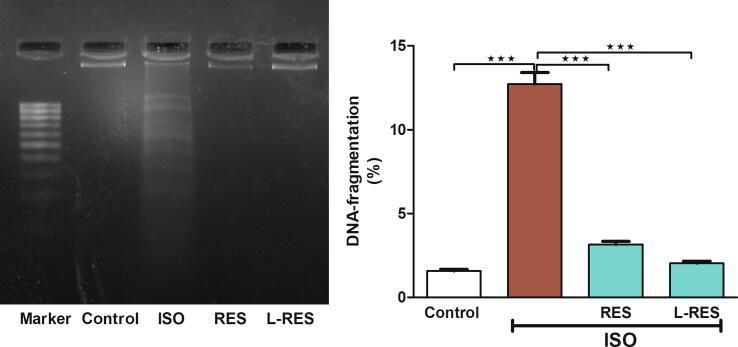
A representative agarose gel image showed variations in the DNA fragmentation in all treated groups, (n = 8). Data were expressed as mean ± SEM. ***P < 0.001.

Analysis for DNA damage revealed a marked DNA fragmentation in the kidney tissues of ISO-intoxicated rats (Fig. 5). Using either RES or L-RES significantly inhibited the degradation of DNA in the kidney tissues, thus restoring the DNA integrity.

### 3.5 RES or L-RES diminished the morphological alterations induced by ISO

**Fig. 6:**
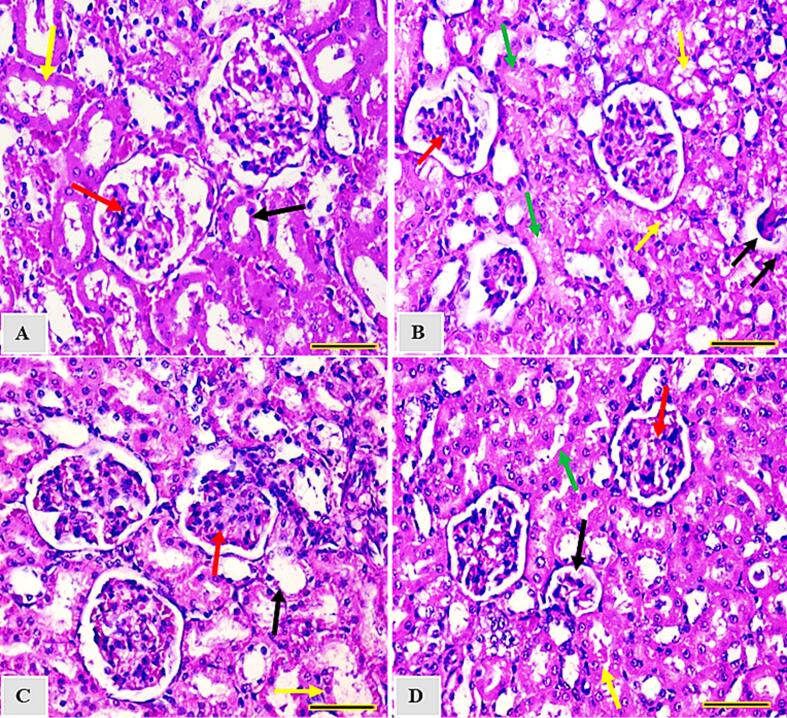
Photomicrographs of hematoxylin and eosin (H&E)-stained sections from kidney of (A) Kidney section from control group showed renal cortex showing renal corpuscle with normal glomerulus (red arrow), normal pattern of proximal convoluted (black arrow) and distal convoluted (yellow arrow) tubules. (B) Kidney section from animal treated with ISO exhibited few of the glomeruli corpuscles that are obliterated and destructed (black arrows) and cellular (hyperplasia of epithelial cells lining the partial layer of Bowman’s capsule) (red arrow). Proximal convoluted tubules show destructed epithelial lining, obliteration and intra-cytoplasmic vacuoles (yellow arrow), obliteration and destructed epithelial lining of distal convoluted tubules (green arrow). (C) Kidney section from animal treated with RES displays renal cortex showing renal corpuscle with mild cellularity (hyperplasia of epithelial cells lining the partial layer of Bowman’s capsule)(red arrow), almost normal proximal convoluted (black arrow) and distal convoluted (yellow arrow) tubules. (D) Kidney section from animal with L-RES showed almost normal glomeruli (corpuscles) (red arrow), few of the corpuscles are obliterated and destructed (black arrows), proximal convoluted almost normal (yellow arrow) and distal convoluted (green arrow) tubules (H&E, x400, scale bar = 100 µm).

The renal tissues of control group exhibited normal glomerulus with normal proximal and distal convoluted tubules (Fig. 6 A). Exposure to ISO caused obliterated and destructed glomerulus with distructed tubules (Fig. 6 B). As shown in Fig. 6 C and D, concurrent treatment of either RES or L-RES with ISO restored almost normal glomeruli with almost normal proximal and distal tubules with more pronounced protective effect of L-RES.

## 4 Discussion

ISO is a non-selective β-adrenoceptor agonist that can induce myocardial infarction, cardiac hypertrophy, or heart failure (Khalifa et al., 2022, Lin et al., 2022, Song et al., 2023), as well as kidney damage (De Ponte et al. 2017), which can develop chronic kidney disease (Chawla et al. 2011). Since the specific therapies for kidney injury are currently very limited, identifying and developing novel and effective approaches is urgently needed. The polyphenolic compound RES occurs naturally in plants and is found primarily in soy, vegetables, and fruits such as grapes and berries (Bertelli and Das 2009). Furthermore, RES has established a wide range of pharmacological effects, including cardioprotection (Alanazi et al. 2020), neuroprotective (Turner et al. 2015), nephroprotective (Kim et al. 2011), antineoplastic (Oi et al. 2010) and antidiabetic (Crandall et al. 2012) effects. RES has many benefits for human health, including antioxidant, anti-inflammatory, and other biological properties (Salehi et al. 2018). It has been revealed that RES reduces renal injury by suppressing oxidative stress and reducing the expression of inflammatory markers such as tumor necrosis factor-α (TNF-α) and IL-6 (Chowdhury et al. 2022). Many in vivo studies have examined whether RES nephroprotection extends to protect against renal injury caused by a variety of drugs such as cisplatin (Osman et al. 2015), doxorubicin (Alhusaini et al. 2022), and gentamicin (Silan et al. 2007). Still, to date, the nephroprotective effect of RES against ISO-induced renal injury has not yet been tackled. Therefore, the present study examined RES and L-RES’s protective effects on ISO-induced kidney injury, focusing on inflammation and apoptosis.

Renal function assessment is crucial in treating patients with kidney disease or pathologies that affect renal function. As part of this study, renal function markers such as creatinine, urea, and uric acid were measured in rat’s serum. We found that serum creatinine, urea, and uric acid levels were significantly increased following ISO administration compared to the control group. While treatments with the RES or L-RES significantly declined all previous distorted parameters compared to the ISO-administred group. Based on these results, it appears that both RES and L-RES reduce ISO-induced kidney injury in rats, thereby improving kidney function. The findings of Xiao et al. (2021) are consistent with our findings, which reported a significant decrease in serum creatinine and uric acid levels in the RES groups compared to the hyperuricemic nephropathy rats (Xiao et al. 2021). Furthermore, our results are consistent with those of a previous study, in which RES treatment improved renal function by lowering urea, uric acid, and creatinine levels in rat model of kidney injury induced by aluminium chloride (Al-Qhtani and Farran 2017).

Additionally, our study also demonstrated the anti-inflammatory effectiveness of RES and L-RES. Research has shown that free radical overproduction triggers an intracellular signaling cascade that facilitates the expression of IL-6 (Alum et al., 2023, Jaishankar et al., 2014). Our study showed a significant elevation in serum level of IL-6 in ISO administred group compared with the control group, indicating the formation of inflammation. The results of this study are consistent with those of the previous investigation, which revealed that IL-6 gene expression was increased in the ISO-treated renal tissues (Odobasic et al. 2007). However, RES and L-RES administration caused a decline in IL-6 in ISO-treated rats. Evidently, these results indicated that RES and L-RES protective mechanisms in rats’ kidneys might be related to the reduction of inflammatory signaling. These findings agree with a study conducted by Lang et al. (2023), who revealed the protective activity of RES against multiorgan injury caused during preeclampsia in rats through inhibition of the cytokines release, including IL-6 and TNF-α (Lang et al. 2023).

Moreover, there is growing evidence that cystatin c regulates inflammatory and apoptosis processes (Zi and Yuekang, 2018). Cystatin c levels rise with decreased kidney function (Dharnidharka et al., 2002, Roos et al., 2007). Based on our western blot analysis, exposure to ISO significantly increased cystatin c protein expression compared to the healthy group. This finding is consistent with a previous study that examined the plasma level of cystatin c in rats exposed to ISO; the results showed a remarkable elevation in cystatin c levels (Gupta et al. 2020). However, when RES or L-RES are administered, the ISO effects on cystatin c are greatly ameliorated by restoring its regular expression levels. A pronounced effect was observed when L-RES was used. The outcomes of our study aligned with a recent study that suggested that RES could alleviate kidney dysfunction induced by advanced glycation end-products via modulating the levels of inflammatory mediators, including cystatin c (Lan et al. 2023).

Apoptosis has also been shown to play a role in the pathological changes associated with kidney injury (Wu et al. 2022). MAPKs belong to a group of serine/threonine protein kinases that regulate a wide range of cellular mechanisms, including transcription, cell differentiation, development, apoptosis, and immunity (Grave et al. 2022). In the present study, we showed that the MAPK significantly upregulated when the rats were injected with ISO. This finding was in line with a study reporting that ISO treatment activated the MAPK signaling pathway due to the excessive generation of ROS by ISO (Li et al., 2023). Our study found that using RES or L-RES concurrently with ISO significantly alleviated the expression level of MAPK protein by restoring the regular level in kidney tissues. Interestingly, L-RES significantly downregulated the expression of MAPK compared to RES treatment. It appears that these results are consistent with those of a previous study in which RES significantly alleviated oxidative stress and downregulated MAPK protein in diabetic rats, contributing to its renoprotective effects (Chang et al. 2011).

Additional investigation of the antiapoptotic effect of RES and L-RES involved in the current study was measuring the expression of Fas gene. This gene is translated to Fas protein, a member of TNF receptor superfamily containing a death domain (Chowdhury et al. 2022). Fas ligand initiates apoptosis by binding to its surface receptor Fas, which is followed by the activation of caspase-8 and eventually the activation of caspase-3, which is considered a death substrate that induces apoptosis (Chowdhury et al. 2022). According to our data, Fas gene expression was significantly upregulated after ISO exposure compared to the control group. High expression of Fas indicates activation of the extrinsic apoptotic pathway. In line with these findings, a study showed that ISO treatment increased the expression of proapoptotic factors, including Fas, Bax, cytochrome *c*, and caspase-3 and 8 (Thangaiyan et al. 2018). However, RES or L-RES significantly improved the ISO effect on Fas by restoring its regular expression level. Additionally, these findings suggested that RES and L-RES may prevent ISO-induced renal apoptosis by activating extrinsic apoptotic pathways. This research has demonstrated for the first time that the use of L-RES with ISO could provide a renoprotective action by improving the Fas expression. Moreover, we quantitatively measured the DNA fragmentation which is used as a biomarker of apoptosis (Majtnerová and Roušar 2018). Besides, gel electrophoresis is one of the most common techniques used to detect the breakage of DNA double strands (Carter et al. 2022). Accordingly, our data revealed that DNA damage was significantly increased in the kidney tissues of ISO-exposed rats. However, we observed that RES and L-RES significantly prevented the DNA damage in kidney tissues. Our previous work revealed the protective activity of L-RES on doxorubicin-induced DNA fragmentation in kidney tissues (Alhusaini et al. 2022).

To further study the renoprotective mechanisms of L-RES effectiveness, we measured the gene expression of lipocalin-2, and to the best of our knowledge, this is the first study that reported the in vivo effect of L-RES on the expression of the renal lipocalin-2 gene. We found that ISO administration caused a significant upregulation in lipocalin-2 mRNA, while RES or L-RES significantly ameliorated ISO effect on this gene by restoring its regular expression level. Lipocalin-2 is considered an ideal biological early marker for acute kidney injury and can predict clinical outcomes (Li et al. 2020). Likewise, Li and colleagues revealed that the high levels of Lipocalin-2 variants were associated with renal dysfunction in humans (Li et al. 2020). Additionally, it has been found that lipocalin-2 was highly expressed in patients with end-stage renal failure (Viau et al. 2010).

Interestingly, the result of histopathological examination of kidney tissues in this study is consistent with the biochemical and molecular data. The glomeruli of ISO-treated rats exhibited marked proliferative changes, including obliterated and destroyed glomeruli capsules, hyperplasia of the epithelial cells lining the partial Bowman’s capsule, and obliteration and destruction of the epithelial cells. These findings are aligned with an earlier study of mouse model with kidney injury induced by ISO (Zhou et al., 2022). However, rats treated with RES or L-RES displayed apparent improvement in the morphological alterations induced by ISO. This result is consistent with our previous study in which we proved the effectiveness of L-RES in restoring the structural integrity and providing a protective effect against doxorubicin-induced kidney injury (Alhusaini et al. 2022). Therefore, This compound has the ability to maintain the structural and functional integrity of the kidneys in ISO-treated groups almost as much as in normal control tissues.

Taken together, the results obtained from this study provided a greater understanding of RES and L-RES and their potential protective functions in the kidney tissues. L-RES showed a noticeable protective action compared with the regular compound because liposome encapsulation could improve the bioavailability and cellular uptake. The liposomes have the ability to protect their contents of RES from digestion, so the contents can be released inside the cells. In conclusion, the present study indicated that L-RES provided a protective efficacy against ISO-induced renal injury superior to its native compound RES. L-RES reduced the excessive inflammatory and apoptotic responses induced by ISO overdose. L-RES prevented kidney injury caused by ISO, most likely by downregulating cystatin c, MAPK, Fas, and lipocalin-2, and that warrants further investigation for its protective activity against kidney injury. In addition, MAPK, Fas, and lipocalin-2 can provide new therapeutic targets for acute kidney failure.

## CRediT authorship contribution statement

**Ahlam M. Alhusaini:** Project administration, Writing – original draft, Writing – review & editing. **Samiyah M. Alshehri:** Validation, Writing – original draft, Writing – review & editing. **Wedad S. Sarawi:** Methodology, Formal analysis, Writing – original draft, Writing – review & editing. **Hanan K. Alghibiwi:** Validation. **Sumayya A. Alturaif:** Validation. **Reema A. Al khbiah:** Methodology. **Shog M. Alali:** Methodology. **Shaikha M. Alsaif:** Validation, Writing – original draft. **Ebtesam N. Alsultan:** Validation. **Iman H. Hasan:** Methodology, Formal analysis.

## Declaration of Competing Interest

The authors declare that they have no known competing financial interests or personal relationships that could have appeared to influence the work reported in this paper.
